# Polymerization Parameters Influencing the QCM Response Characteristics of BSA MIP

**DOI:** 10.3390/bios4020161

**Published:** 2014-06-16

**Authors:** Nam V. H. Phan, Hermann F. Sussitz, Peter A. Lieberzeit

**Affiliations:** Department of Analytical Chemistry, Faculty for Chemistry, University of Vienna, Währinger Straße 38, A-1090 Vienna, Austria; E-Mails: honamd99@yahoo.com (N.V.H.P.); hermann.franz.sussitz@univie.ac.at (H.F.S.)

**Keywords:** molecular imprinted polymer, protein imprinting, polymer optimization, QCM

## Abstract

Designing Molecularly Imprinted Polymers for sensing proteins is still a somewhat empirical process due to the inherent complexity of protein imprinting. Based on Bovine Serum Albumin as a model analyte, we explored the influence of a range of experimental parameters on the final sensor responses. The optimized polymer contains 70% cross linker. Lower amounts lead to higher sensitivity, but also sensor response times substantially increase (to up to 10 h) at constant imprinting effect (signal ratio MIP/NIP on quartz crystal microbalance—QCM). However, by shifting the polymer properties to more hydrophilic by replacing methacrylic acid by acrylic acid, part of the decreased sensitivity can be recovered leading to appreciable sensor responses. Changing polymer morphology by bulk imprinting and nanoparticle approaches has much lower influence on sensitivity.

## 1. Introduction

**Figure 1:**
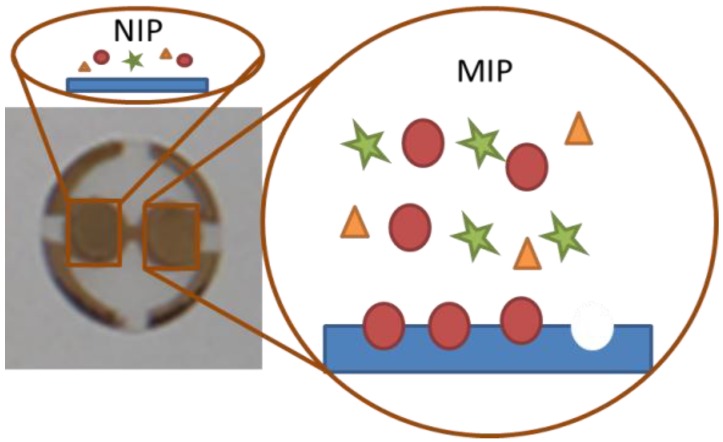
Scheme of a two-electrode quartz coated with two different polymers; molecularly imprinted polymer (MIP) and non-imprinted polymer (NIP).

Recent years have seen very strong increase in everyday application of biotechnology products as well as diagnostics shifting from centralized laboratories to distributed measurements. This also increases the need for quality control and suitable receptors for the respective sensor systems. Natural recognition systems fit perfectly in terms of sensitivity. However, in terms of technological application they are somewhat limited concerning stability and costs. Therefore, artificial recognition systems such as molecularly imprinted polymers (MIP) have attracted increasing attention. These materials aim to mimic natural recognition principles such as antibody-antigen interactions [1,2]. Imprinted polymers are typically cheap, straightforward to generate and effective in creating recognition systems for various types of analytes. Among others, MIP can be combined with mass-sensitive quartz micro balances (QCM) [3] to create powerful sensors for various types of analytes [4,5,6] that can also be applied in real life conditions [7]. Although several imprinting techniques for creating protein MIPs have been developed [8], there is not yet a systematic procedure for protein imprinting. This can be explained by several challenges one faces in protein imprinting [9]: Diffusion of larger bio species into the polymer is inhibited. Proteins can change their steric properties depending on pH and temperature. Due to this fact the steric recognition property of MIP might be lost. Additionally, proteins of course are large molecules containing many functional groups on their surfaces. Depending on the exact orientation during imprinting they can result in a large number of different binding sites on their respective surface area with broadly distributed affinity. Also protein imprinting is mainly limited to aqueous solution, where water competes for the corresponding binding sites with the analyte [10]. Considering this, rationally optimizing those polymers is the first important step on the way to protein MIP sensors. The aim of this paper is to systematically examine various polymer properties, such as cross linker ratio and functional monomer, and their influence on sensitivity and response time. For this purpose Bovine Serum Albumin (BSA) was chosen as model protein. BSA is a globular protein and extensively described in literature [11]. We hence prepared albumin-imprinted polymers for mass-sensitive sensing. All sensor responses of imprinted polymers are compared to a non-imprinted polymer (NIP) to compensate for any physical influence or non-specific adsorption on the surface. As MIP and NIP are chemically identical, any difference in their respective sensor signals only correlates to the imprinting effect. A scheme of such a QCM coated with MIP and NIP, respectively, is shown in Figure 1.

## 2. Experimental Section

### 2.1. Materials and Devices

**Figure 2:**
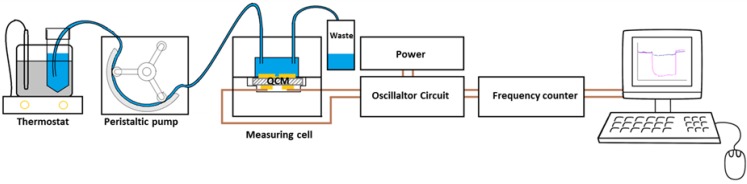
Measurement setup consisting of measuring cell, oscillator circuit and frequency counter. The thermostated solution was pumped through the measuring cell (0.46 mL/min).

Two-electrode 10 MHz QCM were prepared via screen printing of brilliant gold paste (Hereus; 12%) and removing organic residues at 400 °C for 4 h. Afterwards, damping and resonance frequency were determined with an Agilent 8712ET network analyzer. The respective electrodes with a diameter of 5 mm were coated with MIP and NIP and the QCM was mounted into a measurement cell, which was connected to an oscillator circuit. We applied Agilent 53151A frequency counters do determine oscillator frequency as a function of time (the actual sensor signal) except during washing with 0.1 M PBS, 0.1%-SDS and water 10 min each, when read-out was paused. No negative influence of the washing step on the BSA rebinding properties of the sensors could be observed. The overall scheme of this setup is shown in Figure 2. Atomic force microscopy (AFM) measurements took place on a Bruker Instruments Nanoscope 8. Measurements were performed in tapping mode, a TESPA tip was used.

Flow rate required optimization: if chosen too high, noise increases, whereas low flow rate will favor sedimentation and thus lead to false positive signals due to mass change without binding to the selective layer. For these experiments 0.46 mL/min turned out optimal. BSA was purchased at Sigma Aldrich, lyophilized powder ≥98%. All other chemicals were purchased at VWR or Sigma Aldrich in highest available purity. 100 mM PBS Buffer was prepared by diluting 80 g NaCl, 2 g KCl, 14.4 g Na_2_HPO_4_ and 2.4 g KH_2_PO_4_ in 1 L water adjusted to pH 7.4 with HCl. 

### 2.2. Experimental

The general synthetic strategy for obtaining MIP was as follows: First cross linker, functional monomer(s) and the radical starter (potassium peroxidisulfate; 1 mg added per mL monomer solution) were dissolved in 1 mL Water and pre-polymerized under UV light. For the systematic studies of polymers N,N′-(1,2-dihydroxyethylene)bisacrylamide (DHEBA) was used as the cross linker, and methacrylic acid (MAA), acrylic acid (AA) and vinylpyrrolidone (VP) as functional monomers in different ratios. Details of the exact polymer compositions are summarized in Section 3. Depending on polymer composition the translucent solutions turned opaque after approximately 15–30 min indicating successful pre-polymerization. The pre-polymerized solution was spin-coated 5 s with 2000 rpm onto the electrodes. For stamp imprinting we prepared albumin stamps on 5 × 5 mm microscope slides. For that purpose we incubated slides in BSA solution for 1 h at 4 °C. Following that, the BSA stamp was pressed into the oligomer deposited onto one of the QCM electrodes. After hardening for 48 h at room temperature, the stamp was removed by washing with water. In order to remove the template the polymer was washed with 100 mM PBS-Buffer, 0.1%-sodium dodecyl sulphate (SDS) and water 10 min each. In this way we obtained layers that were about 250 nm thick as determined by network analyzer and AFM.

In addition to stamp imprinting we also developed a modified bulk imprinting process. This method has already been described for small molecules [12], however to the best of our knowledge it has not yet been tested on larger bio-species. Pre-polymerization took place as described before but for a minor change: the ingredients were dissolved in 950 µL Water. The oligomer solution was then cooled to room temperature in a water bath, afterwards 50 µL 40 mg/mL Albumin solution was added for the MIP and 50 µL water for the NIP. After 15 min self-organization, oligomer solutions were spin-coated onto the two corresponding electrodes at 2000 rpm for 30 s followed by removing the template from the polymer as described before.

Furthermore, we also prepared MIP nanoparticles. For that purpose, the above mentioned oligomer solutions served to synthesize polymer nanoparticles via precipitation [13]: the solution was added to 15 mL acetonitrile and stirred vigorously for 48 h. The resulting particles were centrifuged, and the supernatant discarded. Finally, the nanoparticles were re-suspended in 1 mL acetonitrile. The concentrated solution was spin-coated onto the gold electrodes at 2000 rpm for 30 s, after 48 h the surface was washed as described before. To determine nanoparticle diameters the solution was diluted 1:10, spin coated onto a glass slide, dried for 24 h at ambient conditions and measured via atomic force microscopy. 

## 3. Results and Discussion

### 3.1. Polymer Optimization: Cross Linker

**Figure 3:**
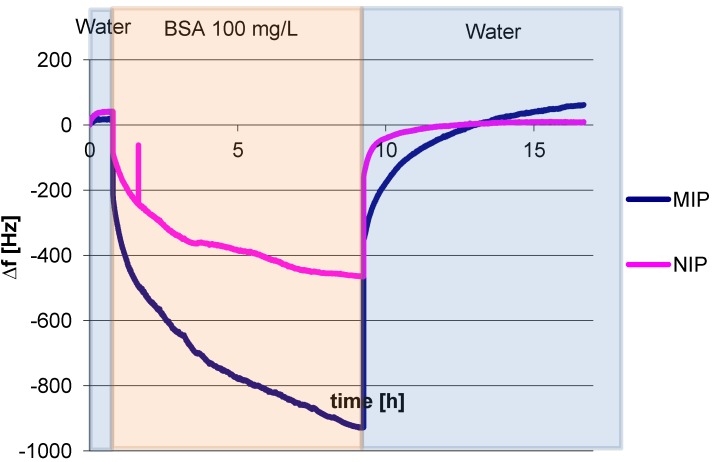
Quartz micro balance (QCM) sensor responses of 100 mg/L albumin.

In a first step we assessed the influence of the cross linker ratio on sensor responses. As a starting point for optimization, we prepared a polymer with a cross linker ratio of 30% (w/w) by mixing 15 mg DHEBA, 25 mg MAA and 10 mg vinylpyrrolidone. One corresponding QCM signal is shown in Figure 3. After equilibration to achieve constant frequency with a noise level around 5–7 Hz, we added a solution of 100 mg/mL albumin in PBS buffer, which leads to decreasing frequency. However, it took 10 h for the signal to reach equilibrium. The frequency difference between 900 Hz for MIP and 500 Hz for NIP corresponds to 400 Hz sensor signal for 100 mg/L Albumin as a consequence of the imprinting process. After washing with water, the measurement was resumed until the signal reached baseline again. Such full reversibility proves both successful washing and full removal of the analyte. In the case of 30% cross linker swelling of the polymer is probable, which usually leads to signal drift, when the signals for both channels change continuously. As reaching stable frequencies could still have lasted for hours without providing further insights, the experiment was stopped. Also at 30% cross linker the mobility of the polymer chain is the highest; therefore some polymer or oligomer can be removed during washing, which explains the slight baseline overshoot in Figure 3.

**Figure 4:**
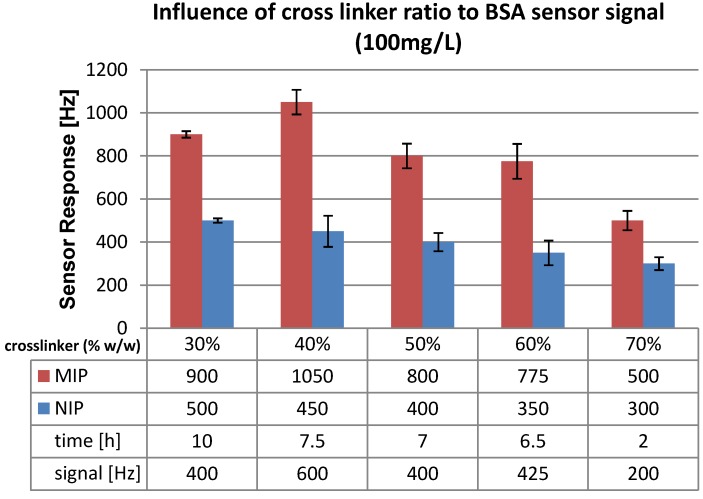
Sensor signals for MIP containing different amounts of cross linker (w/w); functional monomers (MAA:VP; 5:2 w/w).

Repeated measurement with the same quartz revealed that the sensor signal remains basically constant, namely 470 Hz. However the response time increased to 16 h. This effect indicates less pronounced interaction sites, which can be explained by cross-linker cleavage by slightly alkaline washing solutions. During further experiments the monomer ratio (MAA:VP) was kept constant at 5:2 (w/w), while the cross-linker ratio was increased up to 70% (w/w). The corresponding polymer compositions are summarized in Table 1 below. Polymers with cross-linker ratios of 40, 50, 60 and 70% (w/w) were prepared and measured as described before. The respective results are shown in Figure 4. The sensors were characterized in terms of sensor signal and response times.

**Table 1:** Polymer compositions with different amount of cross linker.

Cross-linker (%)	30%	40%	50%	60%	70%
**mg**	15	15	25	30	35
**Monomers (mg)**	35	17.5	25	20	15

The highest sensor signal of 600 Hz was obtained with 40% (w/w) cross-linker, while 50% and 60% cross-linker (w/w) showed similar results to 30%. When the cross-linker ratio is increased from 60 to 70% the sensor signal was reduced by a factor of 2 from 425 to 200 Hz. This seems to contradict results obtained with small molecules, where increase of cross linker leads to an increase in sensitivity [14]. However, the main difference between the two systems is that BSA imprints are only present on the surface of the sensor layer and not the full bulk of the film. Polymers with lower cross linker ratio are expected to swell because of the higher mobility of polymer chains. This leads to gel-like structures for which the Sauerbery equation is no longer fully valid. The slow sensor responses in combination with high sensitivity on both polymers (MIP and NIP) indicate that this may lead to adaptive processes between the film and the BSA solution. 

Furthermore response time strongly depends on the amount of cross-linker: The higher the amount of cross linker, the lower response times become. For instance, when going from 30% to 70% cross-linker, the time for the sensors to reach equilibrium is reduced by a factor of five from 10 to 2 h. This can also be explained by a more rigid polymer where the surface is more clearly defined compared to a soft polymer. Due to increased cross linker ratio, fewer functional monomer binding sites are present on the surface. 

### 3.2. Polymer Optimization: Functional Monomer

A cross-linker ratio of 70% was chosen for further systematic studies of functional monomers to ensure appreciably rapid sensing events. The different polymer compositions for assessing the role of functional monomers are described in detail in Table 2. 

**Table 2:** Polymer compositions with a constant ratio of cross linker (70% w/w).

	Name	cross linker	functional monomer 1	functional monomer 2	functional monomer ratio w/w
**ratio (w/w)**	-	70%	30%		-
		35 mg			
	MA 1	DHEBA	methacrylic acid	vinylpyrrolidone	5:2
	AA 1	DHEBA	acrylic acid	vinylpyrrolidone	5:2
	AA 2	DHEBA	acrylic acid	vinylpyrrolidone	1:1
	AA 3	DHEBA	acrylic acid	vinylpyrrolidone	2:3

**Figure 5:**
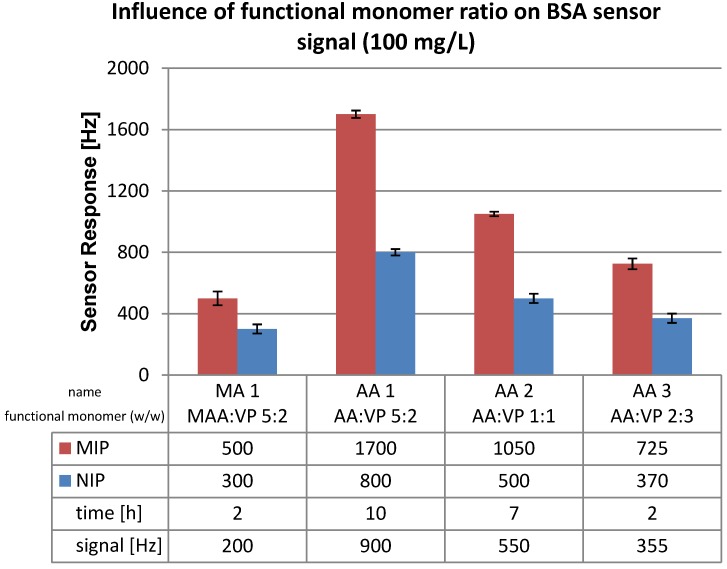
QCM sensor signals obtained for different ratios of functional monomers; cross linker 70%. The sensor signal shown is the difference in response between MIP and NIP.

The outcome for all these materials is summarized in Figure 5. Sensitivity could be increased significantly by using acrylic acid as a monomer rather than methacrylic acid: sensor responses went from 200 Hz to 900 Hz and thus increased by a factor of 4.5. However the response times increased by roughly the same factor. Varying the ratio of the two functional monomers (acrylic acid and vinyl pyrrolidone) has small, but highly significant effects: Functional monomer ratio of 1:1 (w/w) AA:VP leads to a sensor signal of 550 Hz while a ratio 2:3 (w/w) led to 355 Hz. Increasing the amount of vinylpyrrolidone thus reduces sensitivity. However, response times also reached the initial value. 

Compared to the first composition containing 30% cross-linker and 5:2 MAA:VP functional monomer ratio, the sensor signal of 70% cross-linker with 2:3 AA:VP functional monomer is only 1.2 times lower, but the sensor response time decreased by a factor of 2.5. Therefore, the functional monomer ratio of 2:3 AA:VP with 70% cross-linker ratio fits perfectly in terms of sensor response and sensitivity. The selection of imprinting protocols has always relied on balancing different molecular properties such as combination of strong/dissociable (e.g., methacrylic acid) or others on weak/neutral binding monomers (e.g., acrylamide) for template recognition [15].

**Figure 6:**
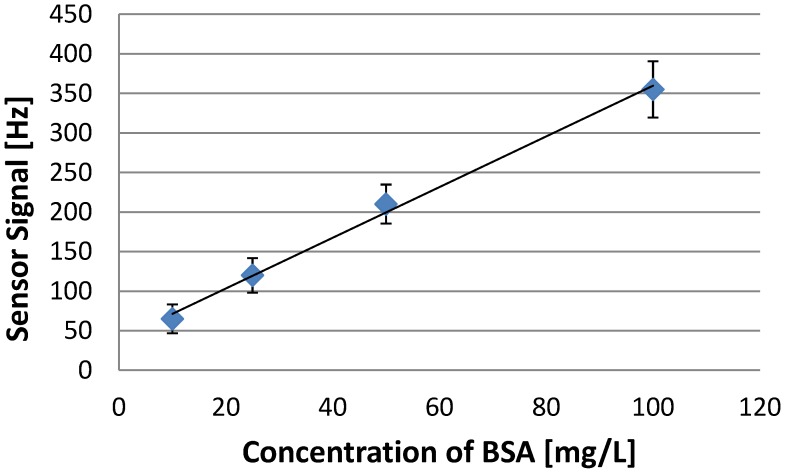
Sensor characteristic of the optimized polymer towards Albumin.

To obtain a sensor characteristic of an Albumin sensor, polymer AA3 with a cross linker ratio of 70% (w/w) and a functional monomer ratio of 2:3 AA:VP was chosen. First measurements indicate that the acrylic acid is the driving force for the interaction between the protein and the polymer. When decreasing the functional monomer ratio of acrylic acid the interaction is reduced, therefore the sensor response signal is decreased. On the other hand the diffusion into the polymer seems to be enhanced by a higher amount of VP. It was possible to calibrate the sensor in a dynamic range ranging from 10 to 100 mg/L (0.15–1.5 µM/L) as shown in Figure 6.

Sensor signals of 65 Hz for 10 mg/L, 120 Hz for 25 mg/L, 210 Hz for 50 mg/L and 355 Hz for 100 mg/L were obtained. The sensor shows a linear behavior. Generally noise is around 5–7 Hz so in theory a signal of 15 Hz could be determined which would mean a (theoretical) limit of detection of 5 mg/L. In Literature LoD of 0.1 mg/L has been reported for Human Serum Albumin [16]. This detection limit is lower than the one for BSA reported here; however, it was obtained by antibody-antigen interaction. In general detection systems derived by nature are often more sensitive than their artificial counterparts, but they cannot be used under nonphysiological conditions and are far more expensive. Artificial recognition systems can hence be a cost-effective alternative in technological applications after optimization.

### 3.3. Polymerization Solvent

It is expected that polar aprotic media would be better for the polymerization process because water competes with the corresponding protein. To investigate the effect the same polymer was prepared in dimethylsulfoxide (DMSO). The sensor signal is reduced by a factor of 1.75 to 200 Hz compared to the same polymer composition prepared in water. Poor solubility of albumin in DMSO hence counteracts the signal increase achieved from optimization.

### 3.4. Polymer Morphology

Molecularly imprinted polymers can be achieved by various strategies, for instance stamp imprinting was chosen for BSA. However this approach does not necessarily ensure optimal self-organization of the analyte and the functional monomers. Bulk imprinting could be used as an alternative. However, proteins are not necessarily suitable for the harsh polymerization procedure at higher temperature or UV-light: Albumin denatures at 60 °C, which is the temperature it would have to withstand during pre-polymerization for one hour. To solve this problem we modified bulk imprinting. This overcomes some disadvantages of stamp imprinting, such as damaging the surface during stamp removal. For the modified bulk imprinting approach we prepared the optimized polymer from 35 mg DHEBA, 8 mg VP and 6 mg AA and polymerized in water following the procedures described in Section 2.1. Both methods, stamp MIP and modified bulk MIP, lead to similar response times—namely 2 h—and sensor signals (at 100 mg/L Albumin, 355 Hz for the stamp and 320 Hz for the modified bulk approach). Since signals (intensity, response time) are not influenced by the chosen polymerization strategy, systematically optimizing polymer composition evidently is the most important step during recognition layer design for QCM sensors.

**Figure 7:**
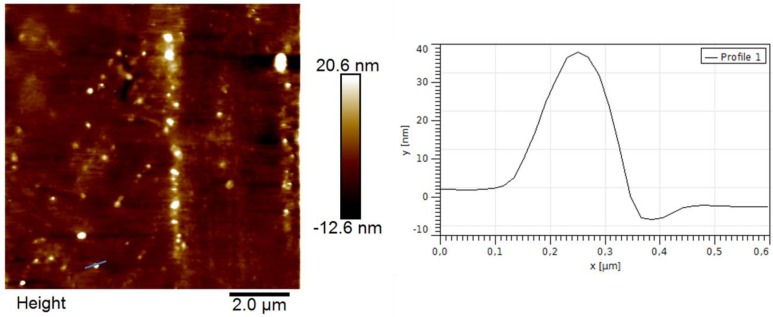
Atomic force microscopy image of BSA MIP nanoparticles.

Polymer morphology also can have some influence on sensor signals, because the number of available binding sites plays an important role for molecular recognition. For instance MIP nanoparticles lead to higher sensitivity in the case of small molecules as analytes due to high surface area and therefore increased amount of accessible binding sites. Nanoparticles are mainly prepared by micro emulsion polymerization [17]. This technique delivers well-defined particles in terms of size and shape. However, the modified bulk imprint approach described earlier makes it possible to create nanoparticles in water by precipitation methods thus substantially reducing the complexity of the polymerization system. Figure 7 shows an AFM image of BSA MIP nanoparticles obtained by such precipitation. Evidently, particles are roughly 350 nm in diameter and seem to be 40 nm thick. The reason for this observation, however, most probably is the fact that soft particles deposited on a surface behave in gel-like manner to form disk-shaped deposits. 

Albumin nanoparticles on QCM revealed a 0.75 times lower signal, namely 277 Hz, compared to MIP thin films resulting from the stamping approach. This can be explained by the large size distribution of the nanoparticles and the geometrical features: As mentioned, NP deposited on the surface are only 30 to 40 nm high and form spots that are 200 to 300 nm in diameter. This indicates that the nanoparticles are flattened during spin-coating. Generally clusters of nanoparticles or flattened particles will exhibit lower surface area than dispersed nanoparticles. Furthermore, the nanoparticles generated in this first attempt could easily be removed from the surface. Hence substantial further effort is necessary to optimize those structures to actually achieve the desired sensor properties.

## 4. Conclusions

These optimization experiments on the way to a standardized stamping procedure for protein MIP revealed substantial influence of the ratio between cross linker and functional monomers: Sensor response time can be decreased by increasing the amount of cross-linker from 30% to 70%. At the same time, also sensitivity decreases, which can be overcome by increasing the polarity of the sensor layer. Replacing methacrylic acid by acrylic acid leads to a higher sensor signal. This clearly shows the importance of optimizing polymer composition. Other parameters play a much smaller role in BSA MIP: replacing water as the polymerization solvent by DMSO, *i.e.*, changing from polar protic to polar aprotic environment, decreases sensitivity despite the fact that this approach should support formation of hydrogen bonds between polymer and template. Polymer morphology also had much smaller influence on the sensor properties than polymer optimization: BSA MIP nanoparticle layers, for instance, lead to slightly lower sensor responses than the corresponding thin films (0.75 times).
